# Weighting of orthostatic intolerance time measurements with standing difficulty score stratifies ME/CFS symptom severity and analyte detection

**DOI:** 10.1186/s12967-018-1473-z

**Published:** 2018-04-12

**Authors:** Alice M. Richardson, Don P. Lewis, Badia Kita, Helen Ludlow, Nigel P. Groome, Mark P. Hedger, David M. de Kretser, Brett A. Lidbury

**Affiliations:** 1 National Centre for Epidemiology and Public Health, Research School of Population Health, ANU, Acton, ACT 2601 Australia; 2 CFS Discovery, Donvale Medical Specialist Centre, Donvale, VIC 3111 Australia; 3 Paranta Biosciences Limited, Melbourne, VIC 3004 Australia; 4 School of Life Sciences, Oxford Brookes University, Headington, Oxford OX3 0BP UK; 5 The Hudson Medical Research Institute, Monash University, Clayton, VIC 3168 Australia; 6 Department of Anatomy and Developmental Biology, Monash University, Clayton, VIC 3168 Australia

**Keywords:** Activins, Pathology, Analytes, Biomarkers, CFS, ME, Standing time, Orthostatic intolerance

## Abstract

**Background:**

Myalgic Encephalomyelitis/Chronic Fatigue Syndrome (ME/CFS) is clinically defined and characterised by persistent disabling tiredness and exertional malaise, leading to functional impairment.

**Methods:**

This study introduces the weighted standing time (WST) as a proxy for ME/CFS severity, and investigates its behaviour in an Australian cohort. WST was calculated from standing time and subjective standing difficulty data, collected via orthostatic intolerance assessments. The distribution of WST for healthy controls and ME/CFS patients was correlated with the clinical criteria, as well as pathology and cytokine markers. Included in the WST cytokine analyses were activins A and B, cytokines causally linked to inflammation, and previously demonstrated to separate ME/CFS from healthy controls. Forty-five ME/CFS patients were recruited from the CFS Discovery Clinic (Victoria) between 2011 and 2013. Seventeen healthy controls were recruited concurrently and identically assessed.

**Results:**

WST distribution was significantly different between ME/CFS participants and controls, with six diagnostic criteria, five analytes and one cytokine also significantly different when comparing severity via WST. On direct comparison of ME/CFS to study controls, only serum activin B was significantly elevated, with no significant variation observed for a broad range of serum and urine markers, or other serum cytokines.

**Conclusions:**

The enhanced understanding of standing test behaviour to reflect orthostatic intolerance as a ME/CFS symptom, and the subsequent calculation of WST, will encourage the greater implementation of this simple test as a measure of ME/CFS diagnosis, and symptom severity, to the benefit of improved diagnosis and guidance for potential treatments.

**Electronic supplementary material:**

The online version of this article (10.1186/s12967-018-1473-z) contains supplementary material, which is available to authorized users.

## Background

Myalgic Encephalomyelitis/Chronic Fatigue Syndrome (ME/CFS) is a clinically defined syndrome characterised by persistent and disabling tiredness leading to functional impairment. Among the case definitions that existed for ME/CFS at the time of this study, the Canadian Criteria [1] was considered to be the most comprehensive. It defined ME/CFS as four or more symptoms, each of which have persisted or recurred during 6 or more consecutive months of illness. These symptoms include: ongoing and unexplained fatigue, post-exertional malaise lasting more than 24 h, and two or more from the definition of Neurological/Cognitive Manifestations (e.g. cognitive or memory impairment), and at least one symptom from two of the following categories, namely, autonomic, neuroendocrine, or immune manifestations. Although contention surrounds the validity of certain criteria, studies utilising this definition have reported a prevalence of between 0.3 and 1.8 percent [2, 3].

The aetiology of ME/CFS is complex and poorly understood. Anecdotally, ME/CFS often begins following an acute episode of “flu-like” symptoms including lymphadenopathy, fever, malaise, headache and sore throat. Such anecdotes are suggestive of infectious agents, particularly viruses. Historically, “clusters” or “outbreaks” of ME/CFS have been frequently linked to known outbreaks of viral illnesses, particularly polio in the 1950s case of the *Royal Free Hospital Group*, or Epstein-Barr virus in the surge of ME/CFS diagnoses in *Lake Tahoe* in the late 1980s [4]. This hypothesis has been empirically investigated by a prospective study of 256 patients diagnosed with Epstein-Barr virus, Q fever or Ross River virus disease, which concluded that 11% of the cohort developed ME/CFS, with similar symptomatology, within 12 months of infection [5]. Current empirical associations exist between ME/CFS and ten different viruses. It is hypothesised that viruses implicated in the pathogenesis of chronic fatigue share two primary features: they cannot be fully eradicated by the immune system, perhaps leading to the chronicity of symptoms, and they can affect the central nervous system, potentially accounting for autonomic dysfunction [6, 7].

It is also important to address the relationship between immune dysfunction and the degree of ME/CFS symptomatology. Unfortunately, most theories of ME/CFS pathogenesis are unable to fully account for the heterogeneity of symptoms experienced, nor the severity of these symptoms. This often leads to the misleading view of ME/CFS as a psychogenic disorder because sufficient histological or biochemical aetiology cannot be identified. Recent research is increasingly identifying sub-groups of ME/CFS populations, who are characterised by dysfunction in particular domains, compared to other patients who also meet the ME/CFS criteria [8, 9].

The standing test is used by clinicians to assess the degree of orthostatic intolerance (OI) in a variety of conditions, including ME/CFS, and for the assessment of cardiovascular response [10, 11], as well as to study of orthostatic hypotension and tachycardia [12]. In addition to time measurements, the standing test can include a subjective score of standing difficulty, which reflects an array of symptoms, for example pre-syncope and pain.

Measures of standing difficulty or pain are often ordinal rather than interval or ratio-scaled due to the subjective and individualised nature of the experience being measured, and in cases where the subject cannot answer for themselves (for example animal clinical scores [13]). The standing time as currently recorded represents a single-category scale with ten levels (2–20 min). There are ordinal pain scales based on pictures, developed for use with pre-numerate children [14].

Weighting or adjusting one measurement by another is well known in the quality of life literature, with *Disability Adjusted Life Years* [15] and *Quality Adjusted Life Years* [16] both being constructed by taking a quantitative measurement (years of life) and multiplying it by a factor less than one that represents the severity of disability or the reduction in quality. Some concerns have been expressed about the value of weighting years of life measurements [17]. While critics do acknowledge that the face validity and intuitive appeal to weighting a rating by an importance, they raise concerns about the high correlation between weighted and unweighted ratings, and the additional complications introduced by multiplying a rating by an importance.

Since the distribution of standing time has not been widely explored, there is value in investigating the distribution of the unweighted standing time in the first instance. Furthermore, it is not yet known whether weighting the standing time could improve the properties of the unweighted standing time, in particular the ability of the standing test to delineate between levels of ME/CFS severity in patients. This study addresses these questions.

## Methods

### Aim

The aim of this paper is to introduce the weighted standing time (WST) as a proxy for ME/CFS severity, involving an Australian cohort recruited from the greater Melbourne region. Questions addressed to achieve this aim comprise the distribution of WST for healthy controls and patients with ME/CFS, the relationship between WST and Canadian criteria for ME/CFS diagnosis, and the relationship between WST and serum/urine analytes collected both for pathology testing required for screening and monitoring patients, as well as serum cytokine profiles.

### Patient cohort

Participants were referred to, or voluntarily presented to, the CFS Discovery Clinic (Donvale, Victoria) between 2011 and 2013. Patients were included if they fulfilled the Canadian Diagnostic ME/CFS criteria [1], and consented to physical examination and biochemical evaluation. Forty-seven consecutive ME/CFS participants were recruited to the study, with two female volunteers excluded from the final cohort due to exceeding the age criteria. One of the remaining 45 patients had a missing standing time measurement (mins), which was estimated using median imputation, allowing the participant to continue in the study. There were 17 control volunteers recruited from the same area of Melbourne, who were in general good health, but not residing with a current patient, nor with a ME/CFS family history. The age range for ME/CFS and healthy control participants was 18–65 years, with a female to male ratio of approximately 4:1.

### Inventories

All research participants (ME/CFS and healthy study controls) completed a full history, physical examination and survey assessment to ascertain their profile under the Canadian Criteria for ME/CFS diagnosis. This generated 48 binary variables across neurological, gut, immune activity, pain and so on, as prescribed by the Canadian Consensus Criteria [1], with each criterion scored as 0 or 1 (absent or present) from the clinical assessment, or questionnaire responses. This study commenced just prior to the publication of the International Criteria [18]; therefore, it continued with diagnosis by the Canadian Criteria for the project duration.

As a minimum diagnostic requirement of ME/CFS, activity limiting fatigue must have been present for at least 6 months, and must have featured post-exertional fatigue (“payback”). A comprehensive profile of additional criteria symptoms was explored, including cognitive and neurological function, immune function, sleep, gastrointestinal function, pain and autonomic responses. Screening symptoms and related physical signs of significance are presented by system in Additional file 1: Table S1. In addition to Canadian Criteria screening, all participants were asked to complete a Depression, Anxiety and Stress Scale (DASS) [19]. No participants were excluded due to DASS results.

### Weighted standing time

The CFS Discovery orthostatic intolerance (standing test) protocol is described in detail elsewhere [10]. Briefly, participants were required to stand, unaided for a maximum of 20 min after a period of repose necessary for baseline (pre-standing) measurements. Heart rate, blood pressure and oxygen saturations were measured at baseline, and subsequently every 2 min during standing. Parameters were measured at the end of the task (either capped at 20 min, or when the participant could no longer continue) and after 3 min of rest following task completion. A difficulty score was also recorded by the nurse, a subjective measure of how difficult the patient found the standing test. A score between 0 and 10 was recorded (0 = no difficulty standing, 10 = support required to stand, pre-syncope). For this study, two further scores were added, with a subjective score of 12 indicating standing difficulty to the point that the standing test was terminated at less than 20 min (but greater than 10 min), and a score of 14 represented the most extreme difficulty where standing was only possible for 10 min, or less.

With the majority of the ME/CFS cohort achieving a standing time of 20 min, comparisons of standing times for ME/CFS and healthy control cohorts were not informative. To weight the standing time in relation to subjective standing difficulty, and produce a single fatigue response variable, the time standing (maximum 20 min, measured at 2 min intervals) and standing difficulty were combined to produce one measure called the “Weighted Standing Time” (WST). The WST (minutes) was calculated by the following equation:

$${\text{Weighted standing time (WST}}) \, = {\text{ Time standing (mins}}) \times (1 - ({\text{Difficulty}}/14)).$$

WST was calculated for each ME/CFS and study control participant, and the WST score (minutes) used thereafter as a measurable proxy for fatigue severity. For statistical analyses, WST was assigned as the response (dependent) variable for comparisons of blood, urine and cytokines.

### Statistical analysis

(i) Clinical (Canadian) criteria—Factor analysis of a tetrachoric correlation matrix (varimax rotation) was conducted to identify significant clinical factors under these diagnostic criteria, as represented by the percentage variance explained by the factor loadings. Tetrachoric correlation allows the analysis of data containing categorical measures, for example “yes” or “no” to the presence of diagnostic symptom under the Canadian Criteria. Analysis of variance was used to determine the statistical difference in factor scores across the WST classes (Table 1).

**Table 1:** Symptoms and related physical signs based on the Canadian Criteria for ME/CFS diagnosis, presented by class, and as assessed at the CFS Discovery Clinic

Canadian criteria symptom classes	Role in CFS/ME diagnosis	Specific symptoms identified	Factor analysis (% variance)* ANOVA (p value)**
Post-exertional malaise and fatigue	Mandatory	Fatigue, malaise and/or pain post effort ≥ 24-h recovery	All symptoms are mandatory (100%)^#^
Minimum 6-months fatigue	Mandatory	≥ 6-months unexplained fatigue	Symptom mandatory (100%)^#^
Sleep disorder	Mandatory	*Difficulty going to sleep* *Sleep during day*	62% (p < 0.0001)
Pain	Mandatory	*Pain* (*any region*) *Headache*	87% (p < 0.0001) 71% (p = 0.118)
Neurological/cognitive manifestations	≥ 2 criteria required	*Light, sound and/or smell sensitivity* *Confusion*	62% (p < 0.0001) 38% (p < 0.0001)
		Autonomic	
		*Breathless*	58% (p < 0.0001)
		*Arrhythmia*	42% (p < 0.0001)
Autonomic/neuroendocrine/immune manifestations	≥ 1 criteria for 2 of the 3 categories required	Gut	
		*Bloating, abdominal pain, diarrhoea*	40% (p < 0.0001)
		Neuroendocrine	
		*Hot/Cold flushes*	73% (p < 0.0001)
		Immune	
		*Tender glands, flulike symptoms, chemical* (*allergy*) *sensitivity*	45% (p < 0.0001)

Identification of significant diagnostic criteria responses via factor analysis of a tetrachoric correlation matrix

Based on Canadian Criteria [1]

* Percentage variance explained, derived from factor analysis of tetrachoric correlation matrix across all CFS/ME severity classes, including healthy study controls

** p values calculated from ANOVA of factor scores across four classes of severity defined by weighted standing time (WST)

^#^No (0%) healthy controls reported a minimum of 6-months fatigue, or post-exertional fatigue/malaise

Factor analyses (FA) were performed using the psych() and lattice() libraries of R3.3.0 [20–22], applying the FA method described by Tabachnick and Fidell [23]. The individual criteria of “Fatigue ≥ 6 months”, “Limited Activity” and “Payback” were omitted from the factor analysis due to 100% of the patients and 0% of the healthy controls reporting their presence. Details of the factors and their loadings are included in Additional file 1: Tables S2–S10.

(ii) Analytes—For the range of blood, urine and cytokine markers compared for ME/CFS versus study (healthy) controls, descriptive statistics were expressed as mean (± standard deviation) for continuous variables, and as proportions (%) for categorical variables. Two sample tests (Mann–Whitney U) were used to determine whether study (healthy) control and ME/CFS participants could be separated statistically at p < 0.05.

(iii) WST severity scale—The WST was used to define three categories of severity for ME/CFS: mild, moderate and severe. Together with the healthy study controls as a distinct WST category, analysis of variance (ANOVA) and follow-up Tukey Highest Significant Difference tests were applied to determine whether statistical significance (p < 0.05) was achieved for individual analytes between the controls and the three levels of ME/CFS severity, as defined by WST (Table 2 and Additional file 1: Table S11).

**Table 2:** Summary statistics for standing time (ST) and weighted standing time (WST) for the ME/CFS and healthy study control groups

Standing criteria (median)	CFS (n = 45) (min)	Healthy (n = 17) (min)	p value
Standing time (IQR)	20 (20–14 = 6)	20 (20–20 = 0)	p = 0.0106^a^
WST (IQR)	10 (14.29–4 = 10.29)	18.57 (14.29–4 = 10.29)	p < 0.0001^a^

^a^Wilcoxon signed rank (WSR) test (p < 0.05)

Because of skewness in both standing time (ST) and weighted standing time (WST) data, Wilcoxon signed rank tests were employed to investigate significance for both median ST and median WST between groups (Table 2). The Wilcoxon signed rank test, Mann–Whitney U, one-way ANOVA and follow-up tests were conducted using R 3.3.0 [20].

Identification of diagnostic criteria that discriminate between categories of ME/CFS were done on the basis that no more than three of the healthy-mild patients recorded presence of the criterion.

### Pathology and cytokine testing

All participant serum, blood and urine analyses were conducted by HealthScope Pathology (now Australian Clinical Laboratories), Clayton Victoria, Australia. Activin proteins and cytokines were measured in serum, as described previously [24–26].

### Human ethics approval

All research participants included in this study provided full, signed consent as dictated by the guidelines of the ANU Human Research Ethics Committee (ANU-HREC). This study and associated protocols were conducted after approval by the ANU-HREC (HREC identification 2011/031).

## Results

### Standing time and WST differ consistently between ME/CFS and study controls

All 17 healthy controls stood for 20 min, with difficulty scores ranging from 0 to 3 (one study control participant had a standing difficulty of 8, but did not fulfil the Canadian criteria for a ME/CFS diagnosis).

Thirty-one (31) of the 45 ME/CFS patients stood for 20 min, with difficulty scores ranging from 1 to 9. The remaining 14 stood for between 2 and 18 min with difficulty scores of 12 or 14, depending on whether standing was achieved for less than or greater than 10 min.

**Fig. 1:**
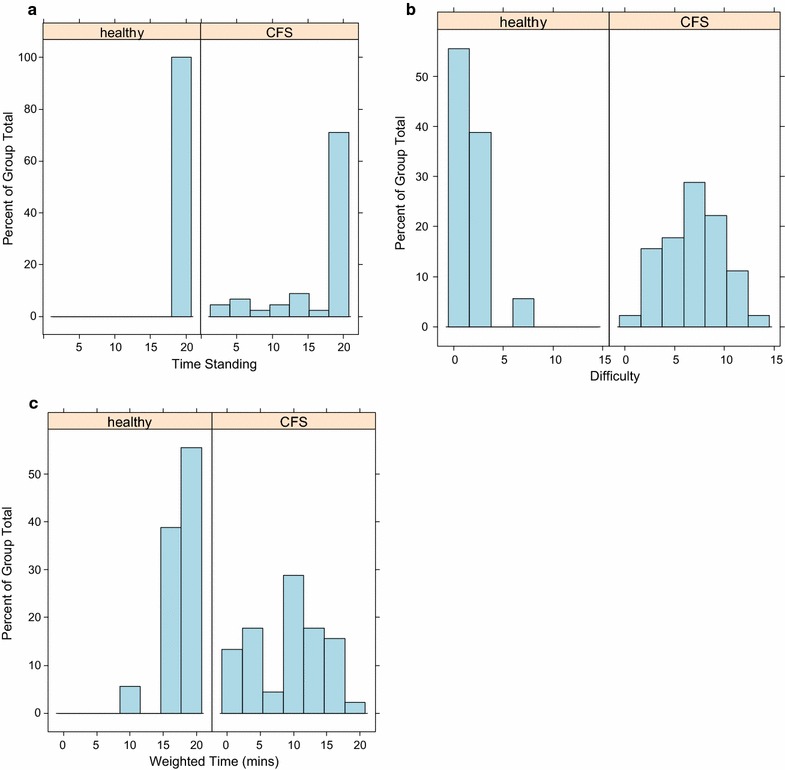
Comparison of raw standing time, standing difficulty profiles and weighted standing time (WST) for ME/CFS and healthy study controls, as performed for the assessment of orthostatic intolerance. **a** Time standing (minutes), **b** difficulty (subjective scale from 0 to 14), and **c** weighted standing time (WST) for 45 CFS patients and 17 healthy study controls

The weighted standing time combines standing time (ST) with a subjective assessment of standing difficulty. The distribution of WST was bimodal for patients, suggesting the existence of moderate and severe degrees of ME/CFS. The distribution of WST for healthy controls is highly skewed with almost no variation (Fig. 1). Wilcoxon signed rank tests (Table 2) showed that there were significant differences for both median ST and WST between ME/CFS and healthy controls.

### WST stratifies ME/CFS severity

The distribution of WST in ME/CFS patients resulted in the assignment of three classes to stratify ME/CFS severity (mild = 1, moderate = 2, severe = 3) as defined by WST (mins), in addition to a class for study controls (healthy = 0). Assessment via the Canadian criteria determined initial assignment as a healthy study control, or ME/CFS participant (see “Methods”).

**Fig. 2:**
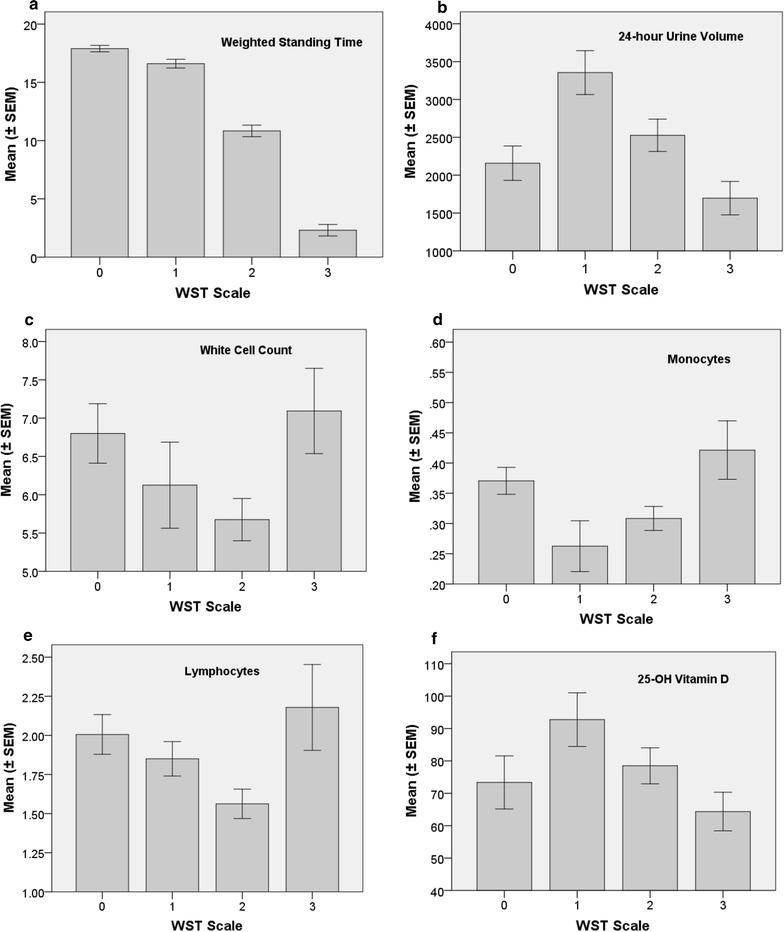
Mean (± SEM) serum, urine and cell marker profiles as summarised by weighted standing time (WST), namely, (**a**) WST (minutes), (**b**) 24-h urine volume (mL/24-h) (**c**) total white cell count (WCC) (× 10^9^/L) (**d**) monocytes (× 10^9^/L) (**e**), lymphocytes (× 10^9^/L) and (**f**) serum Vitamin D (nmol/L). WST classes are: Healthy study controls (0), ME/CFS—mild (1), ME/CFS—moderate (2), and ME/CFS—severe (3) WST class definitions are presented in Table 3

WST differed significantly from class to class for the four categories summarised in Table 3 (F = 188.8; df = 3, 58; p < 0.0001) (and Fig. 2a).

**Table 3:** Definitions of weighted standing time (WST minutes) healthy study controls and ME/CFS classes

Class	n	WST (mins)	Definition of category
No ME/CFS (0)	16	15.0–20.0	Did not meet the Canadian Criteria. All stood 20 min with difficulty ranging from 0 to 3
Mild (1)	8	15.0–20.0	Met the Canadian criteria. All stood 20 min with difficulty ranging from 0 to 3
Moderate (2)	24^a^	6.0–< 15.0	Met the Canadian criteria. All stood 20 min with difficulty ranging from 4 to 10
Severe (3)	14	< 6.0	Met the Canadian criteria. All stood < 20 min with difficulty assigned as 12 or 14

Severe case standing difficulty scored as 12 if the CFS/ME participant stood for 10–18 min, and 14 if standing upright was possible for less than (<) 10 min

^a^Includes 1 healthy control with time = 20 min and difficulty = 8

### Comparison between ME/CFS and healthy study control participant cohorts identified several diagnostic factors from the Canadian Consensus Criteria

There were 48 conditions in the Canadian Criteria [1] that were interrogated by tetrachoric correlation and FA; symptoms that discriminated cases from controls in 100% of cases were not included. Significantly higher proportions of ME/CFS participants consistently reported the presence of symptoms under the Canadian Criteria, except for monthly headache and migraine headache, which were not significantly different between the cohorts. Unexplained fatigue for 6-months or greater (with limited activity), and post-exertional malaise and/or fatigue were mandatory for a positive ME/CFS diagnosis; all ME/CFS research participants had these symptoms, but no healthy study controls. Beyond these mandatory diagnostic criteria, 12 significant Canadian Criteria symptoms (p < 0.0001) were identified by factor analysis (Table 1: specific symptoms in *italics*): Sleep disorders (*Difficulty Going to Sleep, Sleep during Day*), Pain (*Any Body Region, Headache*), Neurological manifestations (*Light/Sound/Smell Sensitivity*, *Confusion*), Autonomic symptoms (*Breathless*, *Arrhythmia*), Gut symptoms (*Bloating/Abdominal Pain/Diarrhoea*), Temperature regulation (*Hot or Cold Flushes*), and Immune symptoms (*Tender Glands/Flu*-*like Symptoms, Chemical Allergy/Sensitivity*).

In terms of the percentage variation in the above symptoms explained by factor analysis, pain (any region of the body) accounted for 87%. Also under the pain factor, headache explained 71% of the symptom variation, but was not significantly different across WST categories as determined via one-way ANOVA (Table 1). The neuroendocrine symptom of poor temperature regulation (*Hot or Cold Flushes*) was also notable explaining 73% of variation across WST categories, as were the neurological/cognitive symptoms of *Light/Sound/Smell sensitivity* at 62%.

Further details on the parameters and implementation of factor analyses (FA) that detected the most predictive clinical criteria are available in Additional file 1: Tables S3–S10.

These results identified specific symptoms within the general clinical domains applied under the Canadian Criteria, and as such, simplified the diagnostic criteria used to assess the patient. With unexplained and persistent fatigue for 6-months or longer, and post-exertional fatigue/malaise as mandatory symptoms, pain (but not headache) and poor body temperature regulation were the leading ME/CFS symptoms identified from the CFS Discovery cohort.

### Clinical criteria discriminate levels of ME/CFS severity, defined by WST

For the non-mandatory Canadian Criteria identified by factor analysis (Table 4), 7–57% of moderate and severe ME/CFS participants reported positively, whereas 13% or fewer healthy controls reported symptoms within these criteria. Therefore, there was a small degree of symptom overlap that will require further differentiation on future investigation.

**Table 4:** Percentage of participants experiencing criteria-defined, non-mandatory symptoms for three levels of ME/CFS severity (increasing order of occurrence from healthy/mild to severe cohorts)

Criterion	Healthy/mild % (n = 24)	Moderate % (n = 21)	Severe % (n = 14)	p value (χ^2^ test)
Headaches monthly	0	14	21	0.0801
Migraine	0	14	7	0.1636
Headaches daily	8	43	28	0.0282*
Sensitive to smell	8	52	57	0.0014*
Sensitive to touch	13	23	36	0.2425
Headaches weekly	13	29	36	0.217

All mild to severe cases reported > 6-months fatigue and post exertional malaise

* Statistical significance at p < 0.05

### Analytes do not differ significantly between ME/CFS and Study Controls, except for serum activin B

From among a broad range of pathology test and cytokine assay results (Additional file 1: Table S2), only serum activin B was significantly different (p < 0.05) for the direct comparison between ME/CFS and study control (healthy) participants, as previously reported [24]. Individual participant serum and urine pathology results generally fell within the laboratory reference interval. Results of investigating the ability of WST stratification to identify significant analyte variation due to orthostatic intolerance severity follow.

### Five routine pathology markers statistically discriminate between levels of ME/CFS severity as defined by WST

One-way ANOVA showed statistically significant differences in values across the four categories of WST (from healthy controls to severe ME/CFS) for five routine pathology markers, namely:

Urine volume (p = 0.0009), URINE CREATININE (p = 0.0092), monocytes (p = 0.0129), lymphocytes (p = 0.0250) and white cell count (p = 0.0381). Serum vitamin D, TSH, anion gap and haemoglobin were not significantly different across the WST scale (Fig. 2 and Additional file: 1).

Figure 2 shows mean WST (± SEM) for several serum or urine markers, for example white cell count and 24-h urine volume. Mean WST is shown for the four levels of ME/CFS severity, to reinforce the discriminatory power of the WST. Post-hoc significant differences between levels of ME/CFS severity are given in Additional file 1: Table S11. The results of other serum markers appear in Additional file 1: Figure S1 since they have broader importance for general fatigue screening (e.g. haemoglobin, TSH, serum vitamin D). Whether statistically significant across the WST severity scale or not, the individual and mean values remained within the specific marker laboratory reference interval for each WST category.

**Fig. 3:**
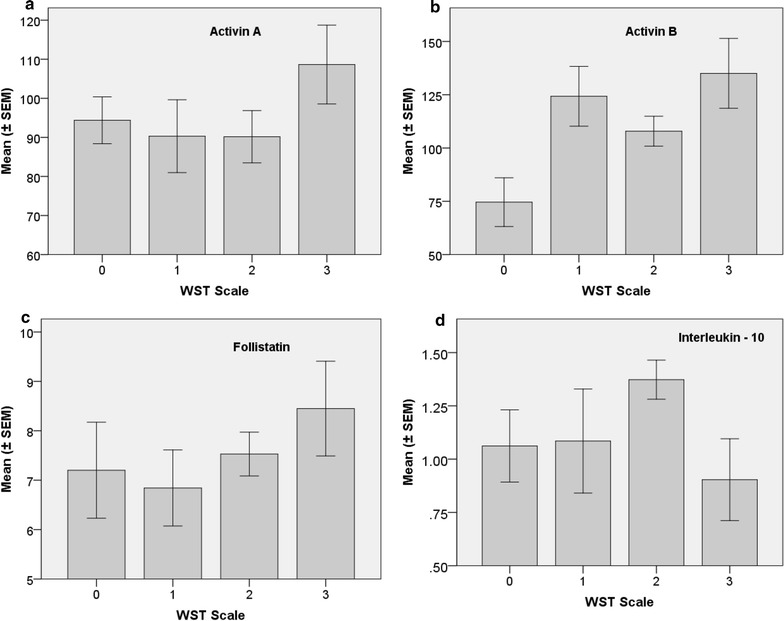
Mean (± SEM) serum concentrations of activin proteins and a representative cytokine as summarised by weighted standing time (WST), namely, (**a**) activin A (pg/mL), (**b**) activin B (pg/mL), (**c**) follistatin (ng/mL), and (**d**) interleukin-10 (IL-10) (pg/mL). WST classes are: Healthy study controls (0), ME/CFS—mild (1), ME/CFS—moderate (2), and ME/CFS—severe (3) WST class definitions are presented in Table 3

Figure 3 summarises the WST-associated mean (± SEM) serum concentrations for activin family proteins (Fig. 3a–c). A statistically significant difference in mean values was detected for activin B (F = 3.549; df = 3, 57; p = 0.02), but not activin A, or the activin-binding protein follistatin. Interleukin 10 (IL-10) did not vary significantly across WST categories (Fig. 3d), and nor did IL-2, IL-4, IL-6 and IFN-γ. TNF and IL-17A were analysed also, but were not detected in the majority of study participant serum samples (results not shown).

## Discussion

This study consisted of two primary participant groups: healthy (study) controls, and patients who satisfied the Canadian criteria for ME/CFS [1]. Comparison of the criteria and analytes in those two groups found that no routine pathology marker was significantly different at p < 0.05, and found no patterns of abnormal results for ME/CFS patients when applying standard reference intervals [24, 27]. The calculation of a weighted standing time (WST), which combined the participant’s time standing (minutes) with a subjective standing difficulty score, measured as a component of orthostatic intolerance (OI) assessments by a specialist ME/CFS clinic with 20 years’ experience. While OI is not unique to ME/CFS, the presence of debilitating fatigue for 6-months, or longer, and post-exertional fatigue/malaise increases its relevance to ME/CFS.

The WST scale, with three levels of ME/CFS severity, allowed the detection of significant variation (p < 0.05) for a number of routine markers via one-way ANOVA. Therefore, this interpretation of pathology data via WST response profile provides a new insight in how to utilize pathology data for ME/CFS diagnosis and monitoring, outside of traditional reference interval observation practices.

The difficulties of diagnosing ME/CFS and delineating its severity are reflected by the twenty-separate clinical/consensus criteria developed over the years, with the 1994 Fukuda criteria the most popular in the research context [28, 29]. This lack of diagnostic consensus can be mitigated by the development of measurable scales, like the WST, associated with easily accessible serum/urine markers that are requested from pathology departments when assessing patients for ME/CFS. Hornig et al. [30] had success in this regard, with serum cytokine profiles separating the ME/CFS cohorts into short-term or long-term patients. IFN-γ was the prominent cytokine marker separating short—from long-term patients, with network and recursive partitioning analyses describing the broader relationships between the immune markers tested, as well as age. Recent investigations of ME/CFS cytokine profiles have shown statistical significance for TGF-β and Resistin when compared to matched healthy controls, as well as significant correlations of seventeen cytokines with disease severity, with thirteen being pro-inflammatory [31].

Attempts to stratify ME/CFS patients into sub-classes has a long history, and past efforts have also included immune responses [32], including in the context of sleep or exercise deprivation [33]. Comorbidity with postural orthostatic tachycardia syndrome (POTS) has been also considered when looking to further classify ME/CFS symptoms, including in terms of neurohumoral and haemodynamic profiles [34]. According to Reynolds et al. [10], patients with comorbid POTS were significantly younger, and thus had symptoms for a shorter period, had greater standing task difficulty, and significant variations in autonomic functioning. These features were identified in 11% of a retrospective cohort comprising 306 CFS Discovery patients, similar to that identified by a British study [35].

Vitamin D deficiency is a well-known outcome for the bed-bound, as is often the case in severe cases of ME/CFS [36]. The observable, but statistically non-significant drop in Vitamin D levels observed in this study as ME/CFS severity increases, suggests that vitamin D deficiency is not a contributor to ME/CFS symptoms, or severity, for this cohort. Likewise, Thyroid Stimulating Hormone (TSH) does not vary significantly between WST categories. Similarly, haemoglobin was not significantly different. In contrast, the statistically significant change in 24-h urine volume for differing levels of ME/CFS severity was observed, consistent with the anomalies in a larger cross-sectional study [27]. IL-10 has been implicated in the pathogenesis of ME/CFS [37, 38], but there is no evidence from this study for a relationship between IL-10 levels and presence/severity of ME/CFS symptoms. On the other hand, Activin B has been recently identified as significant when separating this ME/CFS cohort from the same study (healthy) controls, [24] a relationship that was also reflected by WST analysis.

Factor analysis of ME/CFS symptoms in children [39] identified three factors that were subsequently referred to as phenotypes: musculoskeletal, migraine and sore throat. Our research has identified a larger set of factors, which may be due to the more complex nature of ME/CFS in adults, or the nature of the criteria used to assess ME/CFS in each study. Outside of the mandatory 6-months unexplained fatigue and post-exertional malaise, body pain (not headache) and temperature regulation perturbations explained 87 and 73%, respectively, of variation across the WST spectrum from healthy to severe ME/CFS symptoms (for this study defined as participants who were unable to stand for 20 min).

Strengths of this study emphasise the innovative use of standing time and difficulty to construct the WST, and from there derive the three categories of ME/CFS severity. The richness of the data set, Canadian Criteria responses and predictions associated with routinely available pathology test results, are also strengths in relation to clinical translation. However, the study is limited by its moderate sample size, which particularly limited the acuity of the factor analyses to completely separate clinical criteria. The number of healthy controls is also small. Unpublished analysis from a separate cohort of healthy controls is incomplete at present, but thus far confirms the results in this paper. Additionally, the ST is only run once per patient due to the clinical context of the fatigued patients and the potentially taxing nature of the test. Future research can focus on accessing historical data in order to increase sample sizes, repeating tests where clinically possible, making use of longitudinal data on patients over time, or undertaking an independent validation with a larger cohort across more locations.

## Conclusions

Myalgic Encephalomyelitis/Chronic Fatigue Syndrome is a disabling yet prevalent condition, and its pathophysiology is poorly understood. This study further defined the key symptoms of the syndrome in an Australian sample recruited from the greater Melbourne area of Victoria, as well as identified analyte patterns unique to ME/CFS. Investigations of ME/CFS pathogenesis were conducted through investigation of biochemical, immunological, haematological and endocrine data, with supporting clinical examination and participant survey data utilised.

Importantly we have shown that WST is a useful measure of ME/CFS severity, and easy to implement in clinical environments. Its usefulness could be further enhanced by recording the exact time standing rather than one rounded to the nearest 2 min. A difficulty score recorded every 2 min would also be a very rich source of data, as would a standard rubric for clinicians or patients to record difficulty. The enhanced understanding of the behaviour of the ST and WST from this study will support a greater use of this simple test as a component of ME/CFS assessment, for future diagnosis and treatment.

## Additional file

**Additional file 1.** Additional Tables and Figures.

## Abbreviations

DAS: Depression, Anxiety and Stress Scale
FS: follistatin
IF: interferon
I: interleukin
ME/CFS: Myalgic Encephalomyelitis/Chronic Fatigue Syndrome
OI: orthostatic intolerance
POTS: postural orthostatic tachycardia syndrome
ST: standing time
TNF: tumour necrosis factor
WST: weighted standing time
