# Supplementary material for: Weighting of orthostatic intolerance time measurements with standing difficulty score stratifies ME/CFS symptom severity and analyte detection
Source: J Transl Med. 2018 Apr 12;16:97. doi: 10.1186/s12967-018-1473-z (PMC5898049; doi:10.1186/s12967-018-1473-z)
Supplement: Supplementary file 1 — Additional file 1. Additional Tables and Figures. [file 12967_2018_1473_MOESM1_ESM.docx]

**Additional file**

| 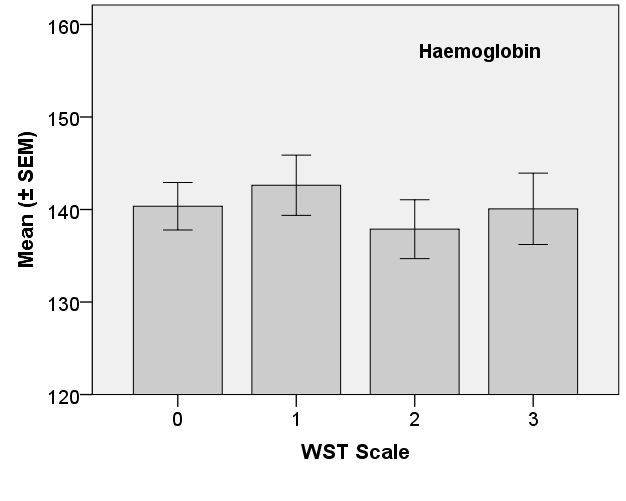 | 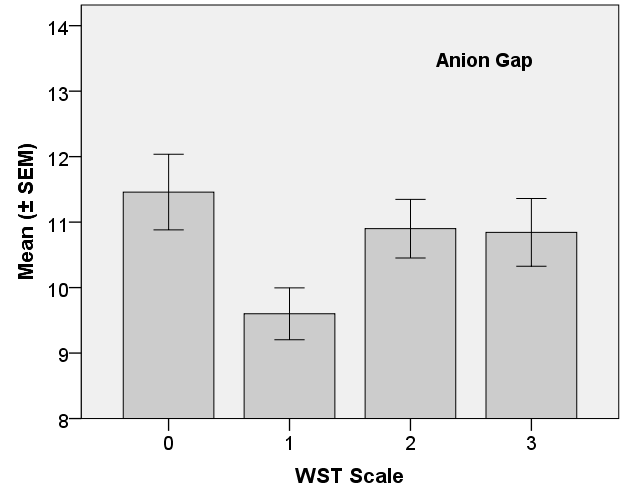 |
| --- | --- |
| 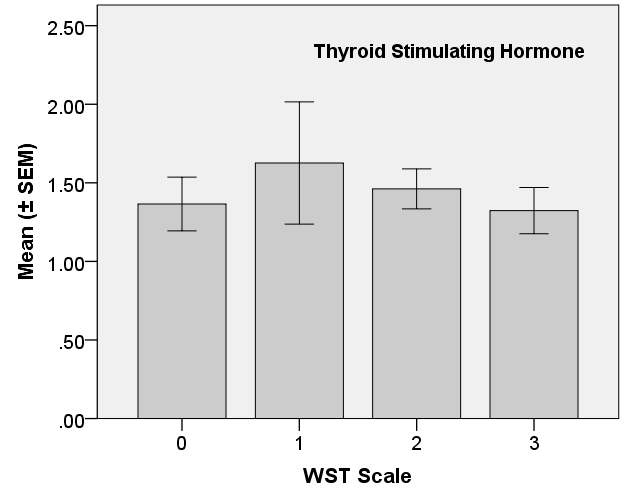 | 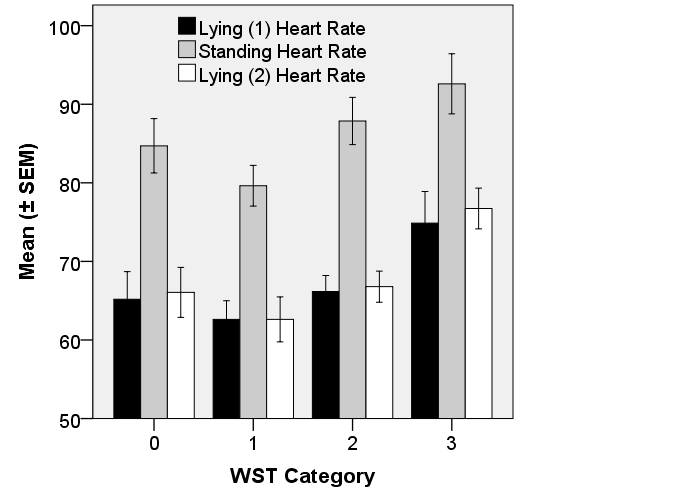 |
| 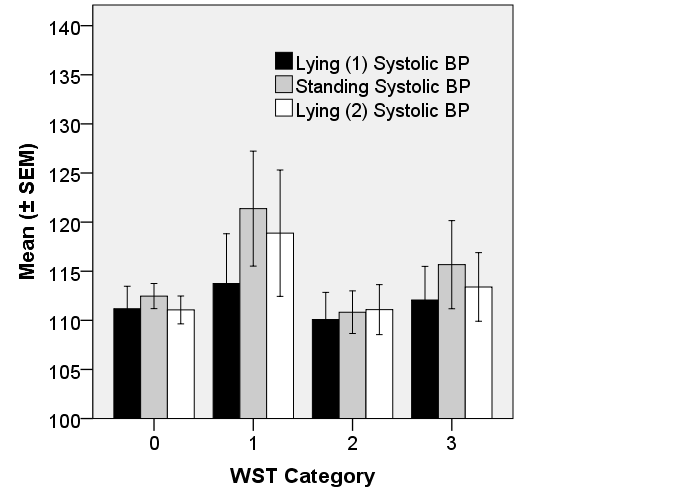 | 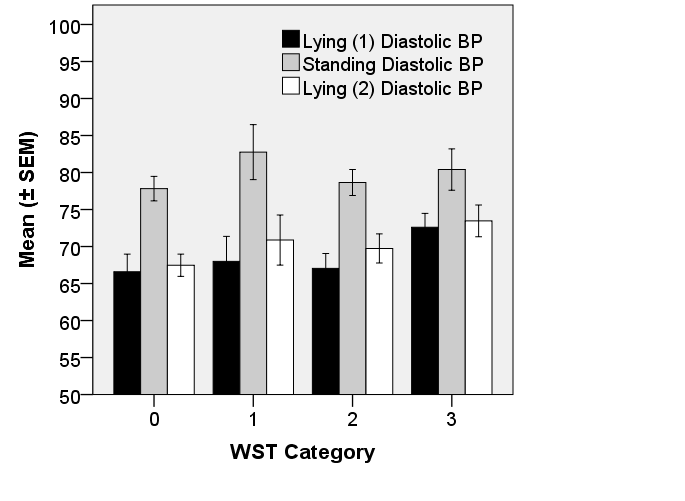 |

**Figure S1** – (a) Haemoglobin (mg/L), (b) Anion Gap (mmol/L), (c) TSH (mIU/L), (d) Heart rate (bpm), (e) systolic blood pressure (mmHg) before (1), during and after (2) the standing test (ST) (f) diastolic blood pressure (mmHg) before (1), during and after (2) the ST, by weighted standing time (WST) category. Figures (a) – (f) represent serum analytes routinely ordered for ME/CFS assessment, to screen for other causes of fatigue. All p > 0.05 (ANOVA).

**Table S1.** Summary statistics (mean and standard deviation) for participant sex, age, pathology and cytokine markers in CFS/ME patients compared to healthy study controls

| **Variable** | **ME/CFS**  **(n = 45)** | **Healthy**  **(n = 17 - 18)** | **p-value^a^** |
| --- | --- | --- | --- |
| Sex | 84% female | 76% female | 0.7814^b^ |
| Age | 37.7 (11.9) | 37.2 (8.7) | 0.909 |
| Haemoglobin | 139.12 (14.22) | 140.89 (10.51) | 0.3650 |
| Red Blood Cells | 4.66 (0.43) | 4.70 (0.31) | 0.3816 |
| Haematocrit | 0.41 (0.04) | 0.42 (0.03) | 0.1761 |
| Mean Cell Volume | 88.84 (5.40) | 90.00 (2.74) | 0.5922 |
| White Cell Count | 6.23 (1.72) | 6.66 (1.67) | 0.4695 |
| Neutrophils | 3.92 (1.25) | 4.11 (1.57) | 0.9272 |
| Lymphocytes | 1.81 (0.72) | 1.98 (0.52) | 0.1770 |
| Monocytes | 0.34 (0.14) | 0.37 (0.09) | 0.1940 |
| Eosinophils | 0.15 (0.10) | 0.18 (0.14) | 0.3342 |
| Platelets | 237.56 (58.05) | 224.11 (38.38) | 0.3454 |
| ESR (inflammatory marker) | 5.93 (5.30) | 4.22 (3.52) | 0.2434 |
| DHEA Sulphate (endocrine hormone) | 4.91 (3.33) | 4.62 (2.09) | 0.7377 |
| Parathyroid Hormone | 4.43 (2.52) | 4.81 (1.90) | 0.4026 |
| Thyroid Stimulating Hormone | 1.45 (0.70) | 1.38 (0.69) | 0.659 |
| Sodium | 139.71 (1.70) | 140.06 (1.39) | 0.7076 |
| Potassium | 4.45 (0.45) | 4.32 (0.27) | 0.4631 |
| Chloride | 104.51 (2.16) | 103.78 (1.40) | 0.2189 |
| Bicarbonate | 29.02 (2.53) | 29.11 (1.32) | 0.9448 |
| Anion Gap | 10.62 (2.00) | 11.49 (2.32) | 0.1779 |
| Urea | 5.01 (1.71) | 5.13 (1.01) | 0.2320 |
| Creatinine | 71.84 (17.51) | 75.11 (6.69) | 0.1778 |
| Glomerular Filtration Rate | 81.20 (9.06) | 81.28 (8.50) | 0.9938 |
| Urate | 0.28 (0.08) | 0.28 (0.06) | 0.7091 |
| Total Protein | 72.67 (3.10) | 73.56 (3.45) | 0.3270 |
| Albumin | 43.00 (2.96) | 42.72 (3.41) | 0.8845 |
| Alkaline Phosphatase (ALP) | 62.42 (22.96) | 55.89 (10.81) | 0.4557 |
| Bilirubin | 10.38 (5.74) | 8.39 (2.83) | 0.3660 |
| Gamma Glutamyl Transpeptidase (GGT) | 17.38 (11.95) | 22.06 (20.43) | 0.1900 |
| AST | 21.11 (4.39) | 22.78 (4.26) | 0.1670 |
| ALT | 20.38 (9.13) | 25.00 (13.28) | 0.2059 |
| Vitamin D | 76.64 (26.79) | 73.61 (32.78) | 0.7320 |
| Fasting Glucose | 4.72 (0.54) | 4.44 (0.34) | 0.1983 |
| Plasma Glucose (0.5-hour post bolus) | 7.65 (1.76) | 7.15 (1.47) | 0.2956 |
| Plasma Glucose (1.0-hour post bolus) | 6.71 (2.39) | 5.98 (2.02) | 0.4270 |
| Fasting Plasma Insulin | 8.14 (4.64) | 8.88 (4.22) | 0.4331 |
| Serum Insulin (0.5-hour post bolus) | 81.33 (43.79) | 50.88 (27.72) | 0.0501 |
| Serum Insulin (1.0-hour post bolus) | 86.92 (54.53) | 94.38 (90.13) | 0.5699 |
| Urine Volume | 2412.8 (1069.7) | 2158.2 (680.5) | 0.6272 |
| Urine Creatinine | 6.41 (3.64) | 6.39 (3.05) | 0.8683 |
| Excreted Creatinine | 11.83 (3.60) | 12.49 (4.56) | 0.9421 |
| Urine Sodium | 60.55 (37.81) | 71.56 (37.98) | 0.3936 |
| Excreted Sodium | 115.86 (68.45) | 142.44 (73.12) | 0.2173 |
| Urine Potassium | 35.11 (18.64) | 33.11 (18.82) | 0.6782 |
| Excreted Potassium | 67.93 (24.26) | 63.89 (18.37) | 0.8370 |
| IL-2 | 0.54 (0.71) | 0.22 (0.50) | 0.0962 |
| IL-4 | 0.55 (0.73) | 0.25 (0.57) | 0.1432 |
| IL-6 | 1.37 (1.31) | 0.87 (1.05) | 0.1992 |
| IL-10 | 1.18 (0.62) | 1.06 (0.66) | 0.4329 |
| FST | 7.72 (2.70) | 7.16 (3.64) | 0.5801 |
| TNF | ND | ND | NA |
| IFN.G | 0.37 (0.72) | 0.57 (0.83) | 0.3148 |
| IL-17A | ND | ND | NA |
| Activin A | 95.74 (34.19) | 94.66 (22.49) | 0.8633 |
| Activin B | 119.45 (46.13) | 76.15 (43.25) | 0.0047 |
| Pre-ST Lying Heart Rate | 68.39 (12.30) | 65.18 (14.55) | 0.2485 |
| Standing Heart Rate | 87.98 (14.06) | 84.71 (14.27) | 0.3447 |
| Post-ST Lying Heart Rate | 69.30 (10.73) | 66.06 (13.11) | 0.2267 |
| Pre-ST Lying Systolic BP | 111.37 (13.22) | 111.18 (9.47) | 0.7566 |
| Standing Systolic BP | 114.24 (14.31) | 112.47 (5.29) | 0.6094 |
| Post-ST Lying Systolic BP | 113.20 (13.76) | 111.06 (5.84) | 0.5054 |
| Pre-ST Lying Diastolic BP | 69.02 (9.10) | 66.59 (9.83) | 0.4111 |
| Standing Diastolic BP | 79.93 (9.53) | 77.82 (6.85) | 0.5505 |
| Post-ST Lying Diastolic BP | 71.15 (9.07) | 67.47 (6.21) | 0.1471 |

a = Mann-Whitney U test. b = two-sample test of proportions.

ND – Not Done due to insufficient sample number (NA – not applicable).

**Factor analysis of the Canadian Criteria**

We used factor analysis [1] to reduce the criteria to a smaller number of independent continuous factors that capture large amounts of the variation in the response to the Canadian criteria.

An exploratory factor analysis was performed using the psych() library of R3.3.0 [2]. The tetrachoric correlation matrix of the Canadian criteria was used as the input because of the binary nature of the Canadian Criteria scores. The software uses ordinary least squares to find the minimum residual factor solution [3]. We used a varimax rotation to enhance interpretability of the rotated factor loadings. We chose the number of factors to retain by inspection of the eigenvalues, retaining no more factors than there were eigenvalues greater than 1.

Because of the number of criteria (53) exceeded the number of patients (45) we extracted loadings for sub-groups of criteria separately. The criteria divide naturally into eight groups, shown in Table S2.

Four patients had missing data in the Canadian criteria. Their values were imputed using median imputation within the fa()function of R3.3.0.

**Table S2.** Eight groups of criteria from the Canadian Criteria for Assessing ME/CFS

| Group | Criteria | Group | Criteria |
| --- | --- | --- | --- |
| Sleep | Difficult Going to Sleep  Restlessness  Difficult to Awake  Sleep during Day | Autonomic | High Heart Rate Upright  Pallor  Arrythmia  Vaso-instability  Sighing Breaths  Breathless |
| Pain | Pain Limits Activity  Pain during Sleep  Pain in all the Body  Pain in Parts of the Body  Pain in Muscles  Pain in Joints | Endocrine | Hot/Cold  Hot/Cold Intolerance  Hypoglycemia  Low Tolerance to Stress |
| Head | Headache Daily  Headache Weekly  Headache Monthly  Migraine | Immune | Tender Glands  Sore Throat  Flu-like Symptoms  New Allergies  Chemical Sensitivity |
| Sensitivity | Sensitive to Light  Sensitive to Smell  Sensitive to Sound  Sensitive to Touch  Ataxia  Overload  Confused | Gut | Bloating  Indigestion  Appetite/Weight Change  Abdominal Pain  Diarrhoea Constipation |

The coefficients of the factor analysis, or loadings, range from -1 to 1. They act as weights to multiply the criteria (0 = absent, 1 = present) to produce a score for each factor for each individual. We applied these scores to the patients to see if the factors were associated with standing time (ST) and weighted standing time (WST), two measures of disease severity.

**Results of the factor analysis**

**Table S3.** Factor analysis of Sleep criteria, with 1 retained factor (eigenvalue = 1.24, proportion of variance explained 62%)

|  | Factor 1 (sleep) |
| --- | --- |
| Difficulty Going to Sleep | 0.50 |
| Sleep during Day | 0.50 |

**Table S4.** Factor analysis of Pain criteria with 1 retained factor (eigenvalue = 1.42, proportion of variance explained 87%)

|  | Factor 1 (pain location) |
| --- | --- |
| Pain in All the Body | 0.65 |
| Pain in Parts of the Body | 0.65 |

**Table S5.** Factor analysis of Head criteria with 1 retained factor (eigenvalue = 1.75, proportion of variance explained 71%)

|  | Factor 1 (pain timing) |
| --- | --- |
| Headache Daily | -0.87 |
| Headache Weekly | 0.87 |

**Table S6.** Factor analysis of Sensitivity criteria with 2 retained factors (eigenvalues 3.42 and 1.52; proportions of variance explained 62% and 38% respectively)

|  | Factor 1 (sensitivity) | Factor 2 (confusion) |
| --- | --- | --- |
| Sensitive to Light | 0.84 | 0.35 |
| Sensitive to Smell | 0.82 | 0.13 |
| Sensitive to Sound | 0.75 | 0.24 |
| Sensitive to Touch | 0.62 | -0.24 |
| Ataxia | 0.53 | 0.35 |
| Overload | 0.13 | 0.62 |
| Confused | 0.09 | 0.93 |

**Table S7.** Factor analysis of Autonomic criteria with 2 retained factors (eigenvalues 1.84 and 0.88; proportions of variance explained 58% and 42% respectively)

|  | Factor 1 (racing) | Factor 2 (irregularity) |
| --- | --- | --- |
| Breathless | 0.84 | 0.04 |
| High Heart Rate Upright | 0.71 | 0.51 |
| Arrhythmias | 0.10 | 0.79 |

**Table S8.** Factor analysis of Endocrine criteria with 1 retained factor (eigenvalue = 1.47, proportion of variance explained 73%)

|  | Factor 1 (hot/cold) |
| --- | --- |
| Hot/Cold | 0.69 |
| Hot/Cold Intolerance | 0.69 |

**Table S9.** Factor analysis of Immune criteria with 2 retained factors (eigenvalues 2.08 and 1.28; proportions of variance explained 45% and 31% respectively)

|  | Factor 1 (flu-like) | Factor 2 (chemical sensitivity) |
| --- | --- | --- |
| Tender Glands | 0.91 | 0.41 |
| Sore Throat | 0.74 | -0.15 |
| Flu-like Symptoms | 0.64 | -0.27 |
| Chemical Sensitivity | -0.09 | 0.99 |

There is some evidence of cross-loading of Tender Glands, but the difference between the 0.91 and 0.41 is large enough for this to be ignored.

**Table S10.** Factor analysis of Gut criteria with 2 retained factors (eigenvalues 2.34 and 0.40; proportions of variance explained 40% and 24% respectively)

|  | Factor 1 (discomfort) | Factor 2 (diarrhoea) |
| --- | --- | --- |
| Bloating | 0.99 | 0.06 |
| Indigestion | 0.71 | -0.06 |
| Appetite/Weight Change | 0.54 | 0.09 |
| Abdominal Pain | 0.44 | 0.78 |
| Diarrhoea | -0.17 | 0.75 |

This factor analysis has demonstrated that ME/CFS is a highly multivariate condition, described by 12 factors across eight facets of the condition.

Factor scores were calculated from the loadings in Tables S3 – S10, and mean scores across the four levels of ME/CFS severity, with 95% confidence intervals, are shown in Figure S2. In all cases a clear distinction between healthy controls and CFS patients can be drawn. The scores do not follow a consistent pattern after that, with some factors scoring lower as ME/CFS severity increase (e.g. autonomic), some scores decreasing then increasing as severity increases (e.g. sleep, pain, headache 1, autonomic 2) and some showing no statistically significant differences as severity increases (e.g. headache 2).

**Figure S2.** Mean factor scores and 95% confidence intervals across the four categories of ME/CFS severity defined by WST.

| **** | **** |
| --- | --- |
| **** | **** |
| **** | **** |
| **** | **** |
| **** | **** |

**Table S11.** Post-hoc analysis of differences in mean blood/serum/urine marker between levels of ME/CFS severity, as defined by Weighted Standing Time (WST).

| Biomarker | p-value^a^ | Post-hoc significant differences |
| --- | --- | --- |
| Weighted Standing Time | < 0.0001 | 2-0 (p = 0), 3-0 (p = 0), 3-0 (p = 0), 2-1 (p = 0),  3-1 (p = 0), 3-2 (p = 0) |
| Volume | 0.0019 | 1-0 (p = 0.0369) , 3-1 (p = 0.0005), 3-2 (p = 0.0405) |
| WCC | 0.0683 | NA |
| Monocytes | 0.0378 | 3-1 (p = 0.0234), 3-2 (p = 0.0375) |
| Lymphocytes | 0.0293 | 3-2 (p = 0.0266) |
| Vitamin D | 0.0742 | NA |
| Acitvin A | 0.4964 | NA |
| Activin B | 0.0217 | 3-0 (p = 0.0033) |
| Follistatin | 0.6417 | NA |
| IL10 | 0.1311 | NA |

a = F test for overall significance between WST groups

**References:**

[1] Tabachnick BG, Fidell LS. Using multivariate statistics, 6th edition. 2013; Boston, MA: Pearson.

[2] R Core Team. R: A language and environment for statistical computing. 2013; Vienna, Austria: R Foundation for Statistical Computing. <http://www.R-project.org/>. Accessed 20 October 2017.

[3] Revelle W. psych: Procedures for Personality and Psychological Research. 2013; Northwestern University, Evanston, Illinois, USA. http://CRAN.R-project.org/package=psych Version = 1.3.2. Accessed 20 October 2017.
